# Convergent Emergence of Glucomannan β-Galactosyltransferase Activity in Asterids and Rosids

**DOI:** 10.1093/pcp/pcae118

**Published:** 2024-10-11

**Authors:** Konan Ishida, Matthew Penner, Kenji Fukushima, Yoshihisa Yoshimi, Louis F L Wilson, Alberto Echevarría-Poza, Li Yu, Paul Dupree

**Affiliations:** Department of Biochemistry, University of Cambridge, Hopkins Building, The Downing Site, Tennis Court Road, Cambridge CB2 1QW, UK; Department of Biochemistry, University of Cambridge, Hopkins Building, The Downing Site, Tennis Court Road, Cambridge CB2 1QW, UK; Center for Frontier Research, National Institute of Genetics, 1111 Yata, Mishima, Shizuoka 411-8540, Japan; Department of Biochemistry, University of Cambridge, Hopkins Building, The Downing Site, Tennis Court Road, Cambridge CB2 1QW, UK; Department of Biochemistry, University of Cambridge, Hopkins Building, The Downing Site, Tennis Court Road, Cambridge CB2 1QW, UK; Department of Molecular Physiology and Biophysics, University of Virginia, Charlottesville, VA 22903, USA; Department of Biochemistry, University of Cambridge, Hopkins Building, The Downing Site, Tennis Court Road, Cambridge CB2 1QW, UK; Department of Biochemistry, University of Cambridge, Hopkins Building, The Downing Site, Tennis Court Road, Cambridge CB2 1QW, UK; Department of Biochemistry, University of Cambridge, Hopkins Building, The Downing Site, Tennis Court Road, Cambridge CB2 1QW, UK

**Keywords:** Cell wall, Enzyme evolution, GT47, Mannan, MBGT, *Solanum lycopersicum*

## Abstract

β-Galactoglucomannan (β-GGM) is a primary cell wall polysaccharide in rosids and asterids. The β-GGM polymer has a backbone of repeating β-(1,4)-glucosyl and mannosyl residues, usually with mono-α-(1,6)-galactosyl substitution or β-(1,2)-galactosyl α-galactosyl disaccharide side chains on the mannosyl residues. Mannan β-galactosyltransferases (MBGTs) are therefore required for β-GGM synthesis. The single MBGT identified so far, *At*MBGT1, lies in glycosyltransferase family 47A subclade VII and was identified in *Arabidopsis*. However, despite the presence of β-GGM, an orthologous gene is absent in tomato (*Solanum lycopersicum*), a model asterid. In this study, we screened candidate *MBGT* genes from the tomato genome, functionally tested the activities of encoded proteins and identified the tomato MBGT (*Sl*MBGT1) in GT47A-III. Interestingly therefore, *At*MBGT1 and *Sl*MBGT1 are located in different GT47A subclades. Furthermore, phylogenetic and glucomannan structural analysis from different species raised the possibility that various asterids possess conserved MBGTs in an asterid-specific subclade of GT47A-III, indicating that MBGT activity has been acquired convergently among asterids and rosids. The present study highlights the promiscuous emergence of donor and acceptor preference in GT47A enzymes. The independent acquisition of the activity also suggests an adaptive advantage for eudicots to acquire β-GGM β-galactosylation and hence also suggests that the disaccharide side chains are important for β-GGM function.

## Introduction

Mannans are a class of polysaccharides conserved from algae to eudicots but with large structural variations. The simplest structure, homomannan, consisting of β-(1,4)-linked mannosyl residues (Man), is found as a storage polysaccharide in plants such as *Aloe vera* and ivory nuts and as a cell wall polysaccharide in some green algae and red algae (Aspinall 1959, Preston 1979, Painter 1983, de O. Petkowicz et al. 2001, Moreira and Filho 2008). Glucomannan, whose backbone is interspersed with β-(1,4)-linked glucose (Glc) and whose mannosyl residues are often acetylated, is also seen as a storage polysaccharide (e.g. konjac and orchid) (Gille and Pauly 2012, He et al. 2017, Voiniciuc 2022). α-Galactosyl (Gal) substitution on C6 of Man is seen in some storage mannans such as in legume seeds (Meier and Reid 1982, McCleary et al. 1985). Acetylated galactoglucomannan (AcGGM) is widespread in the cell walls of woody tissues of vascular plants and is particularly abundant in conifers (Capek et al. 2002, Handford et al. 2003, Goubet et al. 2009, Terrett et al. 2019, Chernova et al. 2020). Recently, a novel type of galactoglucomannan with a repeating Man–Glc disaccharide unit in the backbone was discovered (Yu et al. 2018). This patterned galactoglucomannan can be not only monogalactosylated as found in AcGGM but also di-galactosylated on the Man residues through a β-(1,2)-linkage on α-(1,6)-Gal. This glucomannan is called β-galactoglucomannan (β-GGM) (Yu et al. 2022). β-GGM is widespread in eudicots, being found in the asterid and rosid clades, including *Arabidopsis*, apples, kiwifruit and tomatoes (*Solanum lycopersicum*) (Schröder et al. 2001, Yu et al. 2022), but has not been found in monocots (Ishida et al. 2023).

Mannan structural variation is likely to be related to physiological function in ways that are only partly understood. In algae, homomannan can serve as a major fibrillar component of the cell wall similar to cellulose in land plants (Preston 1979, Painter 1983, Fernández et al. 2012). The replacement of Man backbone residues with Glc and the addition of acetyl groups on the backbone, or branching by α-Gal, are thought to affect the polysaccharide solubility (Scheller and Ulvskov 2010) and interaction with other cell wall components (Yu et al. 2018). It had been thought until recently that the importance of mannans may have decreased in land plant evolution as xyloglucan became more abundant in cell walls (Scheller and Ulvskov 2010). However, mannans are likely to be essential for some aspects of plant physiology since the putative glucomannan synthase mutant *csla7* shows embryonic lethality and defective pollen tube growth (Goubet et al. 2003, 2009). Indeed, challenging the idea of a waning importance of glucomannan through vascular plant evolution is the fact that eudicots acquired the more complex β-GGM during angiosperm evolution (Yu et al. 2022, Grieß-Osowski and Voiniciuc 2023, Ishida et al. 2023). The significance of β-GGM seems to be obscured in many tissues by the presence of xyloglucan (Yu et al. 2022). As there is a substantial gap in our understanding of the relationship between mannan structures and physiological functions, it is essential to expand our knowledge about the biosynthesis of β-GGM.

Three types of transferase enzymes are involved in mannan biosynthesis: CELLULOSE SYNTHASE–LIKE A (CSLA, GT2), which synthesizes the backbone; MANNAN α-GALACTOSYLTRANSFERASE (MAGT, GT34), which adds the first galactosyl side chain residue; and MANNAN *O*-ACETYLTRANSFERASE (MOAT), which adds acetylation (Voiniciuc 2022). For β-GGM synthesis, an additional activity MANNAN β-GALACTOSYLTRANFERASE (MBGT, GT47) is required to complete the β-(1,2)-galactosyl α-galactosyl disaccharide side chains (Yu et al. 2022). Furthermore, the participation of GT106 family putative glycosyltransferase enzymes in mannan production has been identified (Wang et al. 2013, Voiniciuc et al. 2019), but the activity and function remain unclear. The two major types of mannan are synthesized by different combinations of enzymes in *Arabidopsis* (Goubet et al. 2009, Voiniciuc et al. 2015, Yu et al. 2018, 2022, Zhong et al. 2019). AcGGM of primary and secondary cell walls is synthesized by *At*CSLA9, *At*MAGT1 and *At*MOAT1–4. In contrast, β-GGM is synthesized by *At*CSLA2, *At*MAGT1 and, uniquely for β-GGM, the β-galactosyl transferase *At*MBGT1. Among the four mannan synthesis enzymes, CSLA and MOAT are highly conserved in land plants (Liepman et al. 2007, Zhong et al. 2019). However, close orthologs of *At*MAGT1 and *At*MBGT1 are not found in bryophytes and vascular seedless plants (Ishida et al. 2023). Consistent with the genomic/phylogenetic information, mannan α-Gal side chains have not been found in *Physcomitrium patens* (Ye et al. 2022).

β-GGM biosynthesis shares the same GT families as of xyloglucan (Yu et al. 2022, Grieß-Osowski and Voiniciuc 2023). The β-1,4-glucan backbone is synthesized by CELLULOSE SYNTHASE–LIKE C in the GT2 family, and xylosyl side chains are added by xyloglucan xylosyltransferase in the GT34 family (Julian and Zabotina 2022). The addition of the second side chains (equivalent to β-1,2-Gal of β-GGM) is catalyzed by enzymes in GT47A, which is divided into seven subclades (I–VII) (Yu et al. 2022). Interestingly, GT47A has many xyloglucan-modifying enzymes with varying donor preferences, but there is no clear correlation between the activity and their position in GT47A. For example, *At*MUR3 and *At*XLT2 xyloglucan galactosyltransferases locate to GT47A-VI and GT47A-III, respectively, albeit both with galactosyltransferase activities. *Sl*XST xyloglucan arabinofuranosyltransferases and *Cc*XBT and *Vc*XBT xyloglucan β-xylosyltransferases are classified in GT47A-III along with *At*XLT2 (Schultink et al. 2013, Immelmann et al. 2023, Wilson et al. 2023). Two highly similar GT47A enzymes from *P. patens* in GT47A-I showed distinctive activities: XLT2 activity and xyloglucan arabinopyranosyltransferase activities (Zhu et al. 2018). The range of activities suggests that there is relatively frequent evolutionary emergence of new donor preferences in GT47A enzymes that synthesize xyloglucan side chains.

**Fig. 1:**
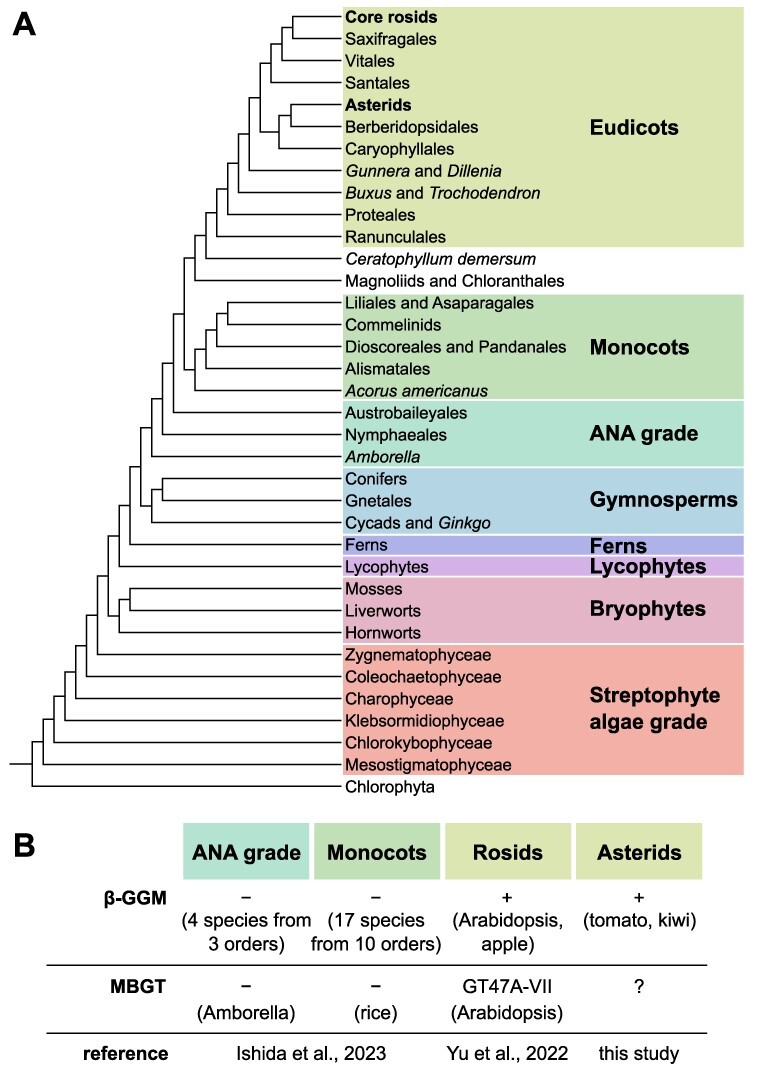
β-GGM and MBGT distribution in the different evolutional stages.

*At*MBGT1 belongs to GT47A-VII and is the only identified MBGT to date (Yu et al. 2022). To investigate conservation of MBGT activity in GT47A-VII in monocots and Amborellales, Nymphaeales and Austrobaileyales (ANA)-grade plants, we previously studied rice (*Oryza sativa*) and Amborella (*Amborella trichopoda*) enzymes (Ishida et al. 2023). Neither candidate exhibits MBGT activity, and the rice GT47A-VII enzyme was shown to be a xyloglucan galactosyltransferase. Moreover, mannans containing β-Gal side chains were not found in more than 20 species of monocot and ANA-grade tissues studied (Ishida et al. 2023) (**Fig. 1**). These findings support the notion that the acquisition of MBGT activity and therefore β-GGM-containing β-Gal side chains is likely to have occurred only in eudicots. However, within eudicots, the evolutionary acquisition of MBGT activity and hence β-GGM is not yet well studied.

(A) Cladogram depicting ancestral relationships between important land plant clades. (B) Summary of the presence or absence of β-GGM and MBGT enzymes in some plant lineages. Asterisks: Genes closest to AtMBGT1 from rice and Amborella did not show MBGT activity in vivo.

Here, we searched for an MBGT enzyme from the asterids, since many asterids synthesize β-GGM. Surprisingly, asterids were found to lack a direct ortholog of *At*MBGT1, and the closest homolog in tomato failed to demonstrate MBGT activity. Instead, based on tomato genomic and transcriptomic data, one candidate was found in GT47A-III, a distant and distinct subclade from that of *At*MBGT1. We showed that the candidate has MBGT activity in vitro and in vivo. Further data support the hypothesis that this MBGT GT47A-III subclade is likely to be specific and conserved throughout asterids. These findings indicate the separate yet convergent acquisition of MBGT activity in GT47 in asterids and rosids, emphasizing the importance of the MBGT-dependent digalactosyl disaccharide side chains for β-GGM functions.

## Results

### Identification of Solyc02g092840 as a candidate asterid MBGT

**Fig. 2:**
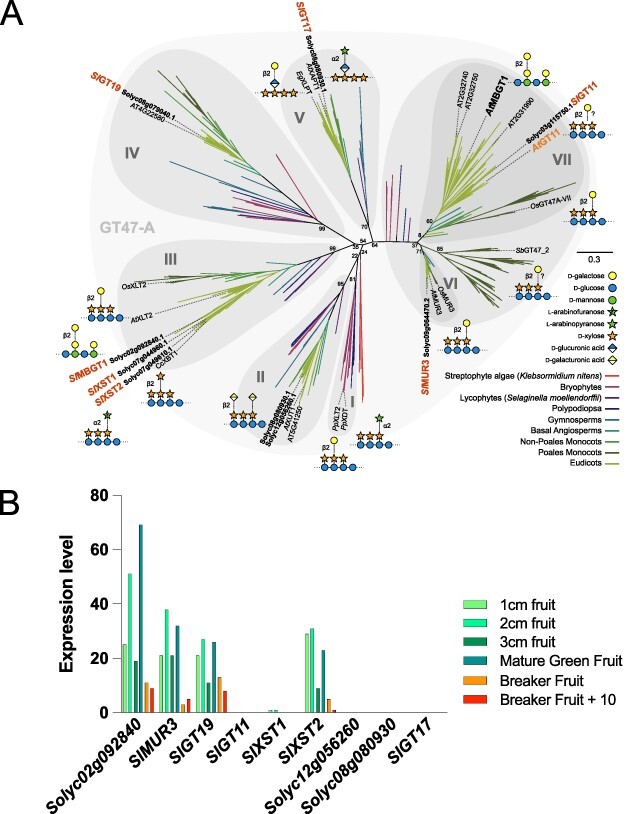
Screening for candidate tomato MBGT.

The MBGT in *Arabidopsis* is in the GT47A-VII clade (Yu et al. 2022). A single GT47A-VII (*Sl*GT11, Solyc03g115750) is found encoded in the tomato genome (**Fig. 2A**). However, its ortholog in *Arabidopsis*, *At*GT11, is thought to be a pollen tube–specific xyloglucan galactosyltransferase (Wei et al. 2021). Therefore, we re-examined the previous phylogeny (Yu et al. 2022) and conducted gene expression analyses to identify any additional MBGT candidates in tomato. Tomato has nine enzymes in the GT47A clade (**Fig. 2A**). Three of them (*Sl*MUR3 and *Sl*XST1,2) have had their activities confirmed by in vivo complementation analysis of the *Arabidopsis* xyloglucan galactose–lacking mutant *mur3-1 xlt2* (Schultink et al. 2013). The other six are *Sl*GT11, two *At*XUT1 orthologs in GT47A-II (Solyc08g080930 and Solyc12g056260), one *At*XLT2 ortholog in GT47A-III (Solyc02g092840), one GT19 ortholog (GT47A-IV; Solyc08g079040) and one GT17 ortholog (GT47A-V; Solyc08g080930). Interestingly, a previous study reported that *Solyc02g092840* did not complement the *mur3-1 xlt2* phenotype (Schultink et al. 2013), raising the possibility that Solyc02g092840 works on a different substrate. In line with this, gene expression data from fruits showed that *Solyc02g092840* was the most highly expressed GT47A gene through fruit development [that β-GGM is abundant in tomato fruits has been shown in our previous paper (Yu et al. 2022)] (**Fig. 2B**). In contrast, other genes encoding potential MBGT candidates, such as *SlGT11, AtXUT1* orthologs and *SlGT17*, had lower expression levels. Hence, we selected Solyc02g092840 as an MBGT candidate.

(A) Phylogenetic tree of the GT47A. Relevant GT47A members from tomato are highlighted with names. The tree is adapted from our previous publication (Yu et al. 2022). MUR3: xyloglucan β-galactosyltransferase. XST: xyloglucan α-arabinofuranosyltransferase. XBT: xyloglucan β-xylosyltransferase. The biochemical activity of *Sl*MUR3, *Sl*XST1 and *Sl*XST2 was confirmed previously (Schultink et al. 2013). (B) Tomato GT47A gene expression data in fruits. Original data were obtained from the public database: BAR ePlant tomato (https://bar.utoronto.ca/eplant_tomato/) and are reads per kilobase per million mapped illumina reads.

**Fig. 3:**
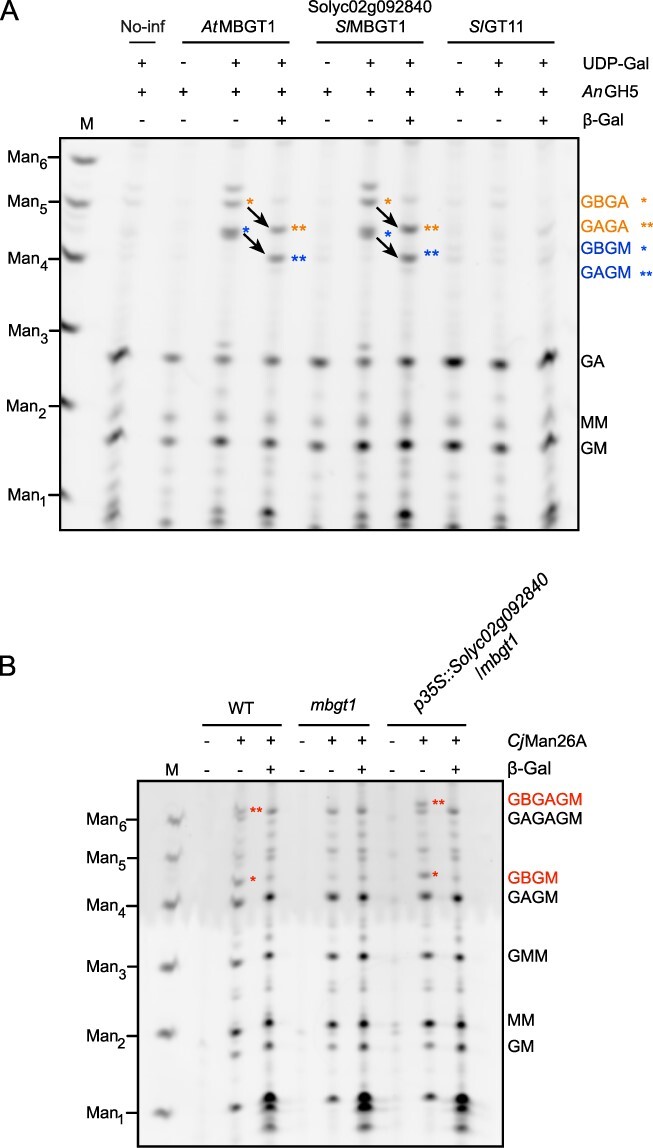
Biochemical activity of Solyc02g092840.

(A) In vitro activity of recombinant Solyc02g092840. No-inf: solubilized microsomal fraction prepared from uninfiltrated tobacco leaves (negative control). After incubation with solubilized microsomes with expressed enzyme and UDP-Gal, substrates were digested with *An*GH5 mannanase and oligosaccharides visualized by PACE. (B) In vivo activity of Solyc02g092840 in the *Arabidopsis mbgt1* background. Cell wall material was digested with *Cj*Man26, β-galactosidase where indicated, and then the oligosaccharides were analyzed by PACE. Three complementation lines were tested, and a representative line is shown. M: marker lane (standard manno-oligosaccharides).

### Solyc02g092840 exhibits MBGT activity

To investigate β-galactosyltransferase activity of Solyc02g092840 in vitro on β-GGM, we transiently expressed the gene *Solyc02g092840* in tobacco (*Nicotiana benthamiana*) leaves. In addition to the candidate, *At*MBGT1 (a positive control) and *Sl*GT11 (the candidate and closest orthologous to *At*MBGT1 in tomato) were also expressed. Microsomal fractions prepared from the transgenic material were subjected to an immunoblot, whereby expression of all three proteins was confirmed (**Supplementary Data S1, Fig. S1**). The microsomal fraction was used as an enzyme source and incubated with UDP-Gal and *Arabidopsis* seed mucilage containing β-GGM as the substrates (mucilage β-GGM lacks β-Gal because of the trimming by β-galactosidases [Voiniciuc et al. 2015, 2016, Yu et al. 2018, 2022)]. The reaction mixtures were treated with *An*GH5 mannanase for structural characterization of the products. Digestion of the initial substrate yields oligos GA and GM [**Fig. 3A**, where A is Gal-α-(1,6)-Man, G is Glc and M is Man] (Yu et al. 2022). Digestion of *At*MBGT1-treated substrate produced the longer oligos including GBGA and GBGM [where B is Gal-β-(1,2)-Gal-α-(1,6)-Man], because the mannanase cleavage is inhibited at β-galactosylated sites. These oligos were sensitive to β-galactosidase and the main products migrated to GAGA and GAGM (**Fig. 3A**). Similar to *At*MBGT1, digestion of Solyc02g092840-treated material also released GBGA and GBGM. In contrast, *Sl*GT11-treated material did not produce β-galactosylated oligos. These results indicate that Solyc02g092840, but not *Sl*GT11, possesses MBGT activity in vitro.

To confirm MBGT activity in vivo, we next performed complementation analysis. *Solyc02g092840* was expressed in the *Arabidopsis mbgt1* mutant under a strong constitutive promoter. The mannan structure in cell walls of young stem from wild-type (WT) (positive control), *mbgt1* (negative control) and complementation lines (*_pro_35S:Solyc02g092840*/*mbgt1*) was analyzed using *Cj*Man26A digestion. This mannanase released larger diagnostic β-GGM-derived oligos distinct from those from digestion of the AcGGM that is also present in the stem cell walls [e.g. MM and GMM, Yu et al. (2022))]. The β-Gal-containing oligosaccharides GBGAGM and GBGM were visible in WT and the complementation line, but these β-galactosidase-sensitive structures were absent in *mbgt1*, which produced only the α-galactosylated oligosaccharides GAGAGM and GAGM (**Fig. 3B**). Together with the in vitro activity evidence, we concluded that *Solyc02g092840* encodes a MBGT that can contribute to the biosynthesis of β-GGM. Thus, we name it *Sl*MBGT1.

### Genes from asterids form a putative MBGT subclade in GT47A-III

The discovery that *Sl*MBGT1 in tomato (an asterid) is in the GT47A-III subclade implies that this enzyme emerged separately from *At*MBGT1 in *Arabidopsis* (a rosid), which resides in the GT47A-VII subclade. We considered whether the GT47A-III group of putative MBGT enzymes might be present more widely in plant lineages beyond tomato. Our previous paper reported asterid enzymes in three subclades within the GT47A-III defined as GT47A-III*a*, GT47A-III*b* and GT47A-III*c* (Wilson et al. 2023). *Sl*MBGT1 is found in the previously uncharacterized subclade GT47A-III*b*. The xyloglucan galactosyltransferases such as AtXLT2 are associated with GT47A-III*a*. Xyloglucan arabinofuranosyltransferases from tomato (*Sl*XST1,2) and cranberry (*Vm*XST1) as well as xyloglucan β-xylosyltransferases from Robusta coffee (*Cc*XBT1) and blueberry (*Vc*XBT) are found in the *c* subclade (Immelmann et al. 2023, Wilson et al. 2023). Based on GT47 protein structural modeling and different activities found in these subclades, the putative donor specificity of GT47A-III subclades was speculated to vary with changes in five regions of amino acid sequence that form the donor sugar binding site (Wilson et al. 2023). Importantly, and in contrast to the XST and XBT sequences, the *Sl*MBGT-containing GT47A-III*b* subclade has greater similarity in these donor site residues to those of the characterized galactosyltransferases, consistent with the notion that the GT47A-III*b* clade consists of enzymes sharing the UDP-Gal donor specificity (Wilson et al. 2023). Genes from multiple orders of the asterid group (Ericales, Cornales, Lamiales, Solanales, Apiales and Asterales) are found in this putative MBGT subclade, suggesting that other asterids may also have MBGT enzymes and have the capacity to synthesize β-GGM.

### Non-Solanales asterids exhibit β-GGM

**Fig. 4:**
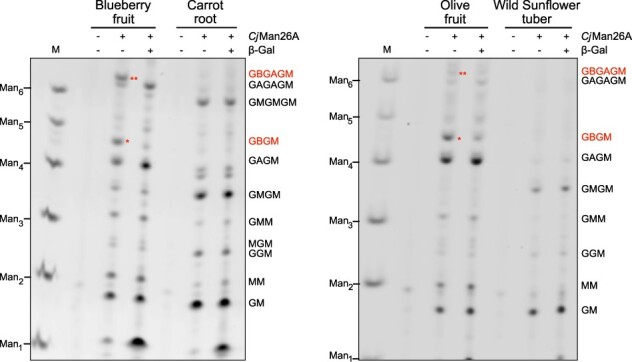
Mannan structure analysis of four species of asterids.

As the phylogenetic analysis suggested the presence of MBGT enzymes in different orders of asterids, these plants should be capable of synthesizing β-GGM. To test this, we collected tissues from blueberry (*Vaccinium corymbosum*, Ericales), carrot (*Daucus carota*, Apiales), olive (*Olea europaea*, Lamiales) and wild sunflower (*Helianthus tuberosus*, Asterales) and analyzed their mannan structures (**Fig. 4**). Coinciding with our prediction, β-galactosylated β-GGM was found in blueberry and olive fruits. Since blueberry is within Ericales, an early branching order of asterids, this suggests that the MBGT activity was already present early in asterid evolution. Interestingly, the subterranean parts from carrot and *H. tuberosus* had unsubstituted β-GGM, which was digested by the mannanase to GM and GMGM. Perhaps the β-GGM in these tissues is trimmed by galactosidases, as seen in *Arabidopsis* seed mucilage β-GGM (Yu et al. 2022). Consistent with the previous identification of β-GGM in kiwifruit (Schröder et al. 2001, Yu et al. 2022), a plant in the asterid Ericales group, this mannan structural analysis suggests that asterid orders possess β-GGM and so have MBGTs, and therefore the other asterid enzymes in the GT47A-III*b* subclade may also have MBGT activity.

Cell wall material was digested with mannanase *Cj*Man26 and β-galactosidase where indicated, and then the oligosaccharides were analyzed by PACE. Blueberries and olive fruits contained β-galactosylated β-GGM; carrot roots and wild sunflower (*H. tuberosus*) tubers contained unsubstituted β-GGM. M: marker lane (standard oligosaccharides).

### Functional convergence may have occurred with different amino acid substitutions

Given that MBGTs originated separately in asterids and rosids, we investigated whether the two distinct GT47 MBGT subclades (GT47A-VII in rosids and GT47A-IIIb in asterids) share the same evolutionary pathway to acquire MBGT activity. If they evolved through a common pathway, convergent amino acid substitutions should be observed at the same protein sites between the two MBGT subclades to allow β-GGM acceptor specificity to replace the xyloglucan acceptor specificity. To test this hypothesis, we performed an amino acid convergence analysis (Fukushima and Pollock 2023). First, based on a phylogenetic tree containing multiple asterid and rosid species, possible asterid and rosid orthogroups with MBGT activity were identified (**Supplementary Data S1 Fig. S2**). In asterids, the orthogroup containing *Sl*MBGT corresponds to the GT47A-III*b* subclade from branchpoint 1605, and we therefore used this as the asterid comparator for the convergence analysis. The rosid orthogroup cannot yet be so precisely defined. We do not know if there is MBGT activity among the *Arabidopsis At*MBGT1 paralogs (AT2G32750, AT2G32740 and AT2G31990), and no other rosid MBGTs have been identified, so it is not yet clear where MBGT activity diverged from the xyloglucan galactosyltransferase activity presumably possessed by the common ancestor with GT47A-VII (**Fig. 2**) (Yu et al. 2022). We therefore examined each of the stem branches between divergence from *Os*GT47A-VII and more recent subclades to investigate any amino acid convergence with the asterid enzymes (**Supplementary Data S1 Fig. S2**). The results did not identify any clear convergence of amino acid sequences that could explain acquisition of the β-GGM acceptor specificity. Thus, distinct amino acid substitutions, or changes that did not occur concurrently with the origins of the subclades, have likely contributed to the convergence of GT47A-III and GT47A-VII activities to MBGTs.

## Discussion

### Recent acquisition of β-GGM

In this study, we identified a tomato MBGT located in GT47A-III, which is distinct from the subclade GT47A-VII to which the rosid *At*MBGT1 belongs. This suggests the functional convergence of GT47A enzymes to acquire MBGT activity and hence also convergence of the β-GGM structure, during eudicot diversification. Furthermore, we found that asterid enzymes form a putative MBGT subclade (GT47A-III*b*) near the XST/XBT subclade (GT47A-III*c*). This subclade is asterid-specific (Yu et al. 2022, Wilson et al. 2023). Therefore, asterids diversified their GT47A-III enzymes after species divergence from other eudicot families. Interestingly, there are similarities in the evolutionary processes that led to MBGT activity in rosids and asterids. In asterids, GT47A-III is likely to have undergone three necessary gene duplication events. At the first divergence in a presumed clade of XLT2 xyloglucan galactosyltransferases, ancestors of XST/XBT/MBGT emerged. Then, the XST/XBT/MBGT ancestor split to create GT47A-III*b* (*Sl*MBGT1 subclade, β-GGM acceptor) and GT47A-III*c* (XST/XBT, xyloglucan acceptor). Interestingly, the rosid MBGT in GT47A-VII may have also evolved from a xyloglucan galactosyltransferase. *At*MBGT1 diverged from the common ancestor with *At*GT11 (GT47A-VII, suggested to be xyloglucan galactosyltransferase) (Wei et al. 2021) and ultimately from a common ancestor with *At*MUR3 (GT47A-VI) xyloglucan galactosyltransferase (**Fig. 2A**). It therefore appears that the acquisition of MBGT activity each time involved a duplication event of genes encoding a xyloglucan galactosyltransferase, and subsequent accumulations of amino acid substitutions allowed the resulting β-galactosyltransferases to accept patterned galactoglucomannan in place of xyloglucan. Despite these similarities, we were unable to identify any convergent changes in amino acid sequence that might suggest critical residues for this change in acceptor binding. Interestingly, XyG fucosylation also appears to have arisen in different GT37 subclades in the course of evolution (Mikkelsen et al. 2021), suggesting that multiple occurrences of selection of GT activity to produce particular plant cell wall polysaccharide structures an important and more general phenomenon.

### Do MBGTs from distinct subclades have different activity?

*At*XLT2 and *At*MUR3 are responsible for galactosylation of different positions on xyloglucan, where *At*XLT2 and *At*MUR3 add galactosyl residues on the second and third positions of a XXXG unit, respectively (Madson et al. 2003, Jensen et al. 2012). Given the two-fold screw conformation of the xyloglucan backbone (Levy et al. 1997), the xylosyl residues of the XXXG unit position alternately on one side to the other of the backbone. In other words, two xylosyl residues of *X*X*X*G are on the opposite face to the one in the middle (X*X*XG), making the backbone appear xylosylated every other glucosyl residue on one side of the polymer. In contrast, the opposite side of the backbone appears xylosylated every four residues. It is therefore likely that *At*XLT2 and *At*MUR3 recognize the frequency of xylosyl residues on XyG and thus bind the xyloglucan substrate very differently to position the correct xylosyl residue for galactosylation. This notion led us to speculate that since the two MBGTs from rosids and asterids inherited their substrate recognition from the different ancestral xyloglucan galactosyltransferases, they might also bind their β-GGM substrates differently. In the case of β-GGM, the α-galactosyl substitutions of mannosyl residues always face just one side of the backbone due to the Glc-Man repeating unit of the backbone. When the mannosyl residues are frequently galactosylated (e.g. GAGAGA), every other mannosyl residue of the backbone would be decorated, which structurally mirrors the side XyG backbone that *At*MUR3 recognizes. Therefore, the MBGT enzymes that emerged in rosids (e.g. *At*MBGT1) could better recognize the frequently galactosylated β-GGM. On the other hand, *Sl*MBGT1, a descendant of XLT2 type galactosyltransferases, may favor the substrate decorated with less α-Gal on the β-GGM backbone such as GMGAGM patterns. Indeed, there seemed to be a difference in substitution patterns of β-GGM between *Arabidopsis* and tomatoes, where *Arabidopsis* showed a similar proportion of GBGA and GBGM, while tomatoes primarily had GBGM with little GBGA (Yu et al. 2022). It would be interesting to investigate further whether the β-GGMs in rosids and asterids have distinct substitution patterns and whether such structural differences change the functionality of β-GGM.

### Open question: what functions do glucomannans with different structures have?

We previously noted that both β-GGM and xyloglucan have structural and functional seminaries (Yu et al. 2022). Here, we studied particularly the acquisition of the β-galactosylated disaccharide side chains in β-GGM, the synthesis of which now appears to have evolved at least twice in eudicots. Some structural variations of β-GGM have now been found, from the relatively highly β-galactosylated form in *Arabidopsis* primary cell wall (Yu et al. 2022) and blueberry fruit to the β-GGM from *Arabidopsis* mucilage that lacks almost all β-galactosylation (Yu et al. 2018) and the unsubstituted form in carrot root and wild sunflower tuber (**Fig. 4**). These structural differences appear to be the result of biosynthetic gene expression variation, different specificity of MAGTs and MBGTs and the production of hydrolases that trim the side chain (e.g. MUM2 β-galactosidase in mucilage biosynthesis), rather than the absence of decoration enzymes in the genomes of these different eudicots. Although these structural variations may provide physiological adaptations, their specific role in growth and development remains to be elucidated. Despite the absence of some aspects of the structure in some organs, the convergent evolution of β-GGM biosynthesis to possess a similar structure in both rosids and asterids strongly suggests that there has been an adaptive advantage to possession of the fully galactosylated β-GGM structure in eudicot cell walls. Why both xyloglucan and β-GGM have evolved this structure is an important outstanding question.

## Materials and Methods

### Plant materials

*Arabidopsis* and tobacco (*N. benthamiana*) were grown at 21°C under 16-h day and 8-h night conditions (Ishida et al. 2023). The *Arabidopsis mbgt1-1* mutant (SALK_065561) was as previously studied (Yu et al. 2022). Blueberry fruits and carrot roots were purchased at a local supermarket. Brined green olives were obtained from a supermarket and used after washing them with extensively with water. *Helianthus tuberosus* was grown in a home garden in Cambridge (UK), and subterranean tubers were harvested in January 2023.

### Mannan structural analysis

Mannan structural analysis was conducted using Polysaccharide Analysis by Carbohydrate gel Electrophoresis (PACE) as described previously (Ishida et al. 2023). Briefly, alcohol-insoluble residues and a 4 M KOH alkali-soluble hemicellulose fraction were prepared. Then, mannan was digested sequentially by *An*GH5 or *Cj*Man26A mannanase and *Aspergillus niger* GH35 β-galactosidase (E-BGLAN, Megazyme, Bray, Ireland). The digestion products were labeled by reductive amination with 8-aminonaphthalene-1,3,6-trisulfonic acid and separated by carbohydrate gel electrophoresis.

### Molecular cloning

Tomato gene expression data are from Tomato Genome Consortium (2012) accessed through the BAR expression browser https://bar.utoronto.ca/eplant_tomato/. For tobacco transient protein expression, the *SlMBGT1* gene was cloned into the pHREAC vector as previously described (structure: CaMV 35S promoter + CDS + TEV + eGFP + 10× His-tag + Nos terminator) (Peyret et al. 2019). *Sl*GT11 (structure: CaMV 35S promoter + CDS + TEV + eGFP + 6× His-tag + Nos terminator) was cloned into a vector containing a P19 suppressor of gene silencing from tomato bushy stunt virus flanked by the CaMV 35S promoter and Nos terminator, which was constructed using a Golden Gate–based system (Engler et al. 2009). The *At*MBGT1-Myc expression construct was prepared previously (Yu et al. 2022). The *SlMBGT1* expression vector for in vivo complementation was constructed with a Golden Gate–based system (Engler et al. 2009), in which the strategy and other cloning parts were the same as described previously (structure: CaMV 35S promoter +CDS + Myc-tag + Nos terminator) (Ishida et al. 2023). The coding sequences of *Sl*GT11 and *Sl*MBGT1 were prepared by a commercial gene synthesis service (Integrated DNA Technologies, Coralville, IA, USA). Nucleotide sequences of primers are described in **Supplementary Data S1, Table S1**.

### Heterologous expression and in vitro assay of MBGT

Seven-week-old tobacco (*N. benthamiana*) leaves were infiltrated with the *Agrobacterium* AGL1 strain harboring the constructs described earlier to express the GTs of interest and were harvested 4 d after infiltration. Microsome preparation for enzyme assays and immunoblotting to confirm expression were performed as described previously (Ishida et al. 2023). For Myc-tagged *At*MBGT1, Ab9106, a rabbit anti-Myc antibody (Abcam, Cambridge, UK), was used. For *Sl*MBGT1 and *Sl*GT11, Ab290 rabbit anti-GFP antibody (Abcam) was used. Goat anti-rabbit IgG-HRP conjugates (Bio-Rad, Hercules, CA, USA) were used to detect primary antibodies. The in vitro assay for MBGT activity in the microsomal membrane fractions using acceptor *Arabidopsis* mucilage β-GGM and donor UDP-Gal was carried out as described except that the reaction time was changed from 5 h to overnight (Yu et al. 2022).

### In vivo complementation

*Arabidopsis mbgt1* (Yu et al. 2022) was transformed by Agrobacterium-mediated floral dip using constructs to test MBGT activity as described previously (Ishida et al. 2023). Transformants were chosen by their seed eGFP signal and kanamycin resistance. Then, genotyping was carried out using primers in **Supplementary Data S1, Table S1**.

### Phylogenetic analysis

The species phylogeny for 24 angiosperm species [ANA-grade: *A. trichopoda, Nymphaea colorata*, Monocots: *O. sativa*, Eudicots Rosids: *Arabidopsis thaliana* (Brassicales), *Brassica rapa* (Brassicales), *Capsella rubella* (Brassicales), *Citrus clementina* (Sapindales), *Eucalyptus grandis* (Myrtales), *Fragaria vesca* (Rosales), *Gossypium hirsutum* (Malvales), *Populus trichocarpa* (Malpighiales), *Theobroma cacao* (Malvales), *Vigna unguiculata* (Fabales), Eudicots Asterids: *D. carota* (Apiales), *Helianthus annuus* (Asterales), *Hydrangea quercifolia* (Rosales), *Lactuca sativa* (Asterales), *Lindenbergia philippensis* (Lamiales), *O. europaea* (Lamiales), *Solanum lycopersicum* (Solanales), *Solanum tuberosum* (Solanales), *Vaccinium darrowii* (Ericales), *Vitis vinifera* (Vitales) and other Eudicots: *Aquilegia coerulea* (Ranunculales)] with sequenced genomes was obtained from timetree.org. The phylogenetic analysis of GT47A-III was performed according to previous work (Fukushima and Pollock 2023). In brief, genes homologous to the *Arabidopsis* GT47A-III were retrieved from the publicly available protein-coding gene sets of the 24 species using TBLASTX v2.9.0 with an *E*-value cutoff of 0.01 and >50% query coverage. In-frame codon sequence alignment was performed using MAFFT v7.455 (https://mafft.cbrc.jp/alignment/software/), ClipKIT v0.1.2 (https://github.com/JLSteenwyk/ClipKIT) and CDSKIT v0.9.1 (https://github.com/kfuku52/cdskit). The gene phylogeny was reconstructed using IQ-TREE v2.0.3 (https://github.com/iqtree/iqtree2) with the general time-reversible nucleotide substitution model and four gamma categories of among-site rate variation, and it was then reconciled with the species tree using GeneRax v1.2.2 (https://github.com/BenoitMorel/GeneRax).

### Amino acid convergence analysis

The reconciled gene tree and the codon alignment were used as inputs for CSUBST v1.1.0 (https://github.com/kfuku52/csubst) to infer the codon substitution history, which was then translated using the standard genetic code to obtain the probabilities of amino acid substitutions, as well as their convergence and divergence between focal branches. The inferred substitutions were mapped to the protein structure of *At*MBGT1 predicted by AlphaFold2 (Q84R16) using the ‘site’ function of CSUBST. Posterior probabilities of convergent or divergent substitutions greater than 0.5 were reported.

## Supplementary Material

pcae118_Supp

## Data Availability

All data supporting the findings of this study are available within the paper and within its **supplementary data** published online.

## References

[R1] Aspinall G.O. (1959) Structural chemistry of the hemicelluloses. *Adv. Carbohyd. Chem*. 14: 429–468. 10.1016/s0096-5332(08)60228-3 13794843

[R2] Capek P., Alföldi J. and Lisková D. (2002) An acetylated galactoglucomannan from *Picea abies* L. Karst. *Carbohydr. Res*. 337: 1033–1037. 12039544 10.1016/s0008-6215(02)00090-3

[R3] Chernova T., Ageeva M., Mikshina P., Trofimova O., Kozlova L., Lev-Yadun S., et al. (2020) The living fossil *Psilotum nudum* has cortical fibers with mannan-based cell wall matrix. *Front. Plant Sci*. 11: 488. 10.3389/fpls.2020.00488 PMC7199214 32411161

[R4] de O. Petkowicz C.L., Reicher F., Chanzy H., Taravel F.R. and Vuong R. (2001) Linear mannan in the endosperm of *Schizolobium amazonicum*. *Carbohydr. Polym*. 44: 107–112.

[R5] Engler C., Gruetzner R., Kandzia R., Marillonnet S. and Peccoud J. (2009) Golden gate shuffling: a one-pot DNA shuffling method based on type IIs restriction enzymes. *PLoS One* 4: e5553. 10.1371/journal.pone.0005553 PMC2677662 19436741

[R6] Fernández P.V., Estevez J.M., Cerezo A.S. and Ciancia M. (2012) Sulfated β-d-mannan from green seaweed *Codium vermilara*. *Carbohydr. Polym*. 87: 916–919. 34663054 10.1016/j.carbpol.2011.06.063

[R7] Fukushima K. and Pollock D.D. (2023) Detecting macroevolutionary genotype–phenotype associations using error-corrected rates of protein convergence. *Nat. Ecol. Evol*. 7: 155–170. 36604553 10.1038/s41559-022-01932-7 PMC9834058

[R8] Gille S. and Pauly M. (2012) O-acetylation of plant cell wall polysaccharides. *Front. Plant Sci*. 3: 12. 10.3389/fpls.2012.00012 PMC3355586 22639638

[R9] Goubet F., Barton C.J., Mortimer J.C., Yu X., Zhang Z., Miles G.P., et al. (2009) Cell wall glucomannan in Arabidopsis is synthesised by CSLA glycosyltransferases, and influences the progression of embryogenesis. *Plant J*. 60: 527–538. 19619156 10.1111/j.1365-313X.2009.03977.x

[R10] Goubet F., Misrahi A., Park S.K., Zhang Z., Twell D. and Dupree P. (2003) AtCSLA7, a cellulose synthase-like putative glycosyltransferase, is important for pollen tube growth and embryogenesis in Arabidopsis. *Plant Physiol*. 131: 547–557. 12586879 10.1104/pp.014555 PMC166831

[R11] Grieß-Osowski A. and Voiniciuc C. (2023) Branched mannan and xyloglucan as a dynamic duo in plant cell walls. *Cell Surf*. 9: 100098. 10.1016/j.tcsw.2023.100098 PMC9900609 36756196

[R12] Handford M.G., Baldwin T.C., Goubet F., Prime T.A., Miles J., Yu X., et al. (2003) Localisation and characterisation of cell wall mannan polysaccharides in *Arabidopsis thaliana*. *Planta* 218: 27–36. 12844268 10.1007/s00425-003-1073-9

[R13] He C., Wu K., Zhang J., Liu X., Zeng S., Yu Z., et al. (2017) Cytochemical localization of polysaccharides in *Dendrobium officinale* and the involvement of *Do*CSLA6 in the synthesis of mannan polysaccharides. *Front. Plant Sci*. 8: 173. 10.3389/fpls.2017.00173 PMC5306395 28261235

[R14] Immelmann R., Gawenda N., Ramírez V. and Pauly M. (2023) Identification of a xyloglucan beta-xylopyranosyltransferase from *Vaccinium corymbosum*. *Plant Direct*. 7: e514. 10.1002/pld3.514 PMC10368651 37502316

[R15] Ishida K., Ohba Y., Yoshimi Y., Wilson L.F.L., Echevarría-Poza A., Yu L., et al. (2023) Differing structures of galactoglucomannan in eudicots and non-eudicot angiosperms. *PLoS One* 18: e0289581. 10.1371/journal.pone.0289581 PMC10735049 38127933

[R16] Jensen J.K., Schultink A., Keegstra K., Wilkerson C.G. and Pauly M. (2012) RNA-seq analysis of developing Nasturtium seeds (*Tropaeolum majus*): identification and characterization of an additional galactosyltransferase involved in xyloglucan biosynthesis. *Molecular Plant* 5: 984–992. 22474179 10.1093/mp/sss032 PMC3440008

[R17] Julian J.D. and Zabotina O. (2022) Xyloglucan biosynthesis: from genes to proteins and their functions. *Front. Plant Sci*. 13: 920494. 10.3389/fpls.2022.920494 PMC9201394 35720558

[R18] Levy S., Maclachlan G. and Staehelin L.A. (1997) Xyloglucan sidechains modulate binding to cellulose during in vitro binding assays as predicted by conformational dynamics simulations. *Plant J*. 11: 373–386. 9107029 10.1046/j.1365-313x.1997.11030373.x

[R19] Liepman A.H., Nairn C.J., Willats W.G.T., Sørensen I., Roberts A.W. and Keegstra K. (2007) Functional genomic analysis supports conservation of function among cellulose synthase-like a gene family members and suggests diverse roles of mannans in plants. *Plant Physiol*. 143: 1881–1893. 17307900 10.1104/pp.106.093989 PMC1851810

[R20] Madson M., Dunand C., Li X., Verma R., Vanzin G.F., Caplan J., et al. (2003) The *MUR3* gene of Arabidopsis encodes a xyloglucan galactosyltransferase that is evolutionarily related to animal exostosins. *Plant Cell* 15: 1662–1670. 12837954 10.1105/tpc.009837 PMC165408

[R21] McCleary B.V., Clark A.H., Dea I.C.M. and Rees D.A. (1985) The fine structures of carob and guar galactomannans. *Carbohydr. Res*. 139: 237–260.

[R22] Meier H. Reid J.S.G. (1982) Reserve polysaccharides other than starch in higher plants. *In* Plant Carbohydrates I: Intracellular Carbohydrates. Edited by Loewus, F.A. and Tanner, W. pp. 418–471. Springer, Berlin Heidelberg.

[R23] Mikkelsen M.D., Harholt J., Westereng B., Domozych D., Fry S.C., Johansen I.E. et al. (2021) Ancient origin of fucosylated xyloglucan in charophycean green algae. *Commun. Biol*. 17: 754. 10.1038/s42003-021-02277-w PMC8211770 34140625

[R24] Moreira L.R.S. and Filho E.X.F. (2008) An overview of mannan structure and mannan-degrading enzyme systems. *Appl. Microbiol. Biotechnol*. 79: 165–178. 18385995 10.1007/s00253-008-1423-4

[R25] Painter T.J. (1983) Structural evolution of glycans in algae. *Pure Appl. Chem*. 55: 677–694.

[R26] Peyret H., Brown J.K.M. and Lomonossoff G.P. (2019) Improving plant transient expression through the rational design of synthetic 5ʹ and 3ʹ untranslated regions. *Plant Methods* 15: 108. 10.1186/s13007-019-0494-9 PMC6749642 31548848

[R27] Preston R.D. (1979) Polysaccharide conformation and cell wall function. *Annu. Rev. Plant Physiol*. 30: 55–78.

[R28] Scheller H.V. and Ulvskov P. (2010) Hemicelluloses. *Annu. Rev. Plant Biol*. 61: 263–289. 20192742 10.1146/annurev-arplant-042809-112315

[R29] Schröder R., Nicolas P., Vincent S.J.F., Fischer M., Reymond S. and Redgwell R.J. (2001) Purification and characterisation of a galactoglucomannan from kiwifruit (*Actinidia deliciosa*). *Carbohydr. Res*. 331: 291–306. 11383899 10.1016/s0008-6215(01)00046-5

[R30] Schultink A., Cheng K., Park Y.B., Cosgrove D.J. and Pauly M. (2013) “The Identification of two arabinosyltransferases from tomato reveals functional equivalency of xyloglucan side chain substituents. *Plant Physiol*. 163: 86–94. 23893172 10.1104/pp.113.221788 PMC3762667

[R31] Terrett O.M., Lyczakowski J.J., Yu L., Iuga D., Franks W.T., Brown S.P., et al. (2019) Molecular architecture of softwood revealed by solid-state NMR. *Nat. Commun*. 10: 4978. 10.1038/s41467-019-12979-9 PMC6823442 31673042

[R32] Tomato Genome Consortium (2012) The tomato genome sequence provides insights into fleshy fruit evolution. *Nature* 485: 635–641. 22660326 10.1038/nature11119 PMC3378239

[R33] Voiniciuc C. (2022) Modern mannan: a hemicellulose’s journey. *New Phytol*. 234: 1175–1184. 35285041 10.1111/nph.18091

[R34] Voiniciuc C., Dama M., Gawenda N., Stritt F. and Pauly M. (2019) Mechanistic insights from plant heteromannan synthesis in yeast. *Proc. Natl. Acad. Sci. U.S.A*. 116: 522–527. 30584101 10.1073/pnas.1814003116 PMC6329948

[R35] Voiniciuc C., Günl M., Schmidt M.H.-W. and Usadel B. (2015) Highly branched xylan made by IRREGULAR XYLEM14 and MUCILAGE-RELATED21 links mucilage to Arabidopsis seeds. *Plant Physiol*. 169: 2481–2495. 26482889 10.1104/pp.15.01441 PMC4677919

[R36] Voiniciuc C., Zimmermann E., Schmidt M.H.-W., Günl M., Fu L. and North H.M. (2016) Extensive natural variation in Arabidopsis seed mucilage structure. *Front. Plant Sci*. 7: 803. 10.3389/fpls.2016.00803 PMC4894908 27375657

[R37] Wang Y., Mortimer J.C., Davis J., Dupree P. and Keegstra K. (2013) Identification of an additional protein involved in mannan biosynthesis. *Plant J*. 73: 105–117. 22966747 10.1111/tpj.12019 PMC3558879

[R38] Wei Q., Yang Y., Li H., Liu Z., Fu R., Feng H., et al. (2021) The xyloglucan galactosylation modulates the cell wall stability of pollen tube. *Planta* 254: 133. 10.1007/s00425-021-03779-x 34821984

[R39] Wilson L.F.L., Neun S., Yu L., Tryfona T., Stott K., Hollfelder F., et al. (2023) The biosynthesis, degradation, and function of cell wall β-xylosylated xyloglucan mirrors that of arabinoxyloglucan. *New Phytol*. 240: 2353–2371. 37823344 10.1111/nph.19305 PMC10952531

[R40] Ye Z.-H., Zhong R. and Degola F. (2022) Cell wall biology of the moss *Physcomitrium patens*. *J. Exp. Bot*. 73: 4440–4453. 35348679 10.1093/jxb/erac122

[R41] Yu L., Lyczakowski J.J., Pereira C.S., Kotake T., Yu X., Li A., et al. (2018) The patterned structure of galactoglucomannan suggests it may bind to cellulose in seed mucilage. *Plant Physiol*. 178: 1011–1026. 30185440 10.1104/pp.18.00709 PMC6236596

[R42] Yu L., Yoshimi Y., Cresswell R., Wightman R., Lyczakowski J.J., Wilson L.F.L., et al. (2022) Eudicot primary cell wall glucomannan is related in synthesis, structure, and function to xyloglucan. *Plant Cell* 34: 4600–4622. 35929080 10.1093/plcell/koac238 PMC9614514

[R43] Zhong R., Cui D. and Ye Z.-H. (2019) Evolutionary origin of O-acetyltransferases responsible for glucomannan acetylation in land plants. *New Phytol*. 224: 466–479. 31183872 10.1111/nph.15988

[R44] Zhu L., Dama M. and Pauly M. (2018) Identification of an arabinopyranosyltransferase from *Physcomitrella patens* involved in the synthesis of the hemicellulose xyloglucan. *Plant Direct*. 2: e00046. 10.1002/pld3.46 PMC6508525 31245712

