# Supplementary material for: Convergent Emergence of Glucomannan β-Galactosyltransferase Activity in Asterids and Rosids
Source: Plant Cell Physiol. 2024 Oct 11;65(12):2030–9. doi: 10.1093/pcp/pcae118 (PMC11662442; doi:10.1093/pcp/pcae118)
Supplement: pcae118_Supp [file pcae118_supp.zip › suppl_data/pcp-2024-e-00145-File006.docx]

**Convergent emergence of Glucomannan β-galactosyltransferase activity in Asterids and Rosids**

Konan Ishida^1^, Matthew Penner^1^, Kenji Fukushima^2^, Yoshihisa Yoshimi^1^, Louis F.L. Wilson^1,3^, Alberto Echevarría-Poza^1^, Li Yu^1^, Paul Dupree^1*^

**Affiliations:**

1: Department of Biochemistry, University of Cambridge, Hopkins Building, The Downing Site, Tennis Court Road, Cambridge, UK

2: Center for Frontier Research, National Institute of Genetics, 1111 Yata, Mishima, 411-8540, Shizuoka, Japan

3: Department of Molecular Physiology and Biophysics, University of Virginia

*Corresponding author: [pd101@cam.ac.uk](mailto:pd101@cam.ac.uk)

**Corresponding author:**

P. Dupree

Department of Biochemistry, University of Cambridge, Hopkins Building, The Downing Site, Tennis Court Road, Cambridge, UK

Supplementary data S1


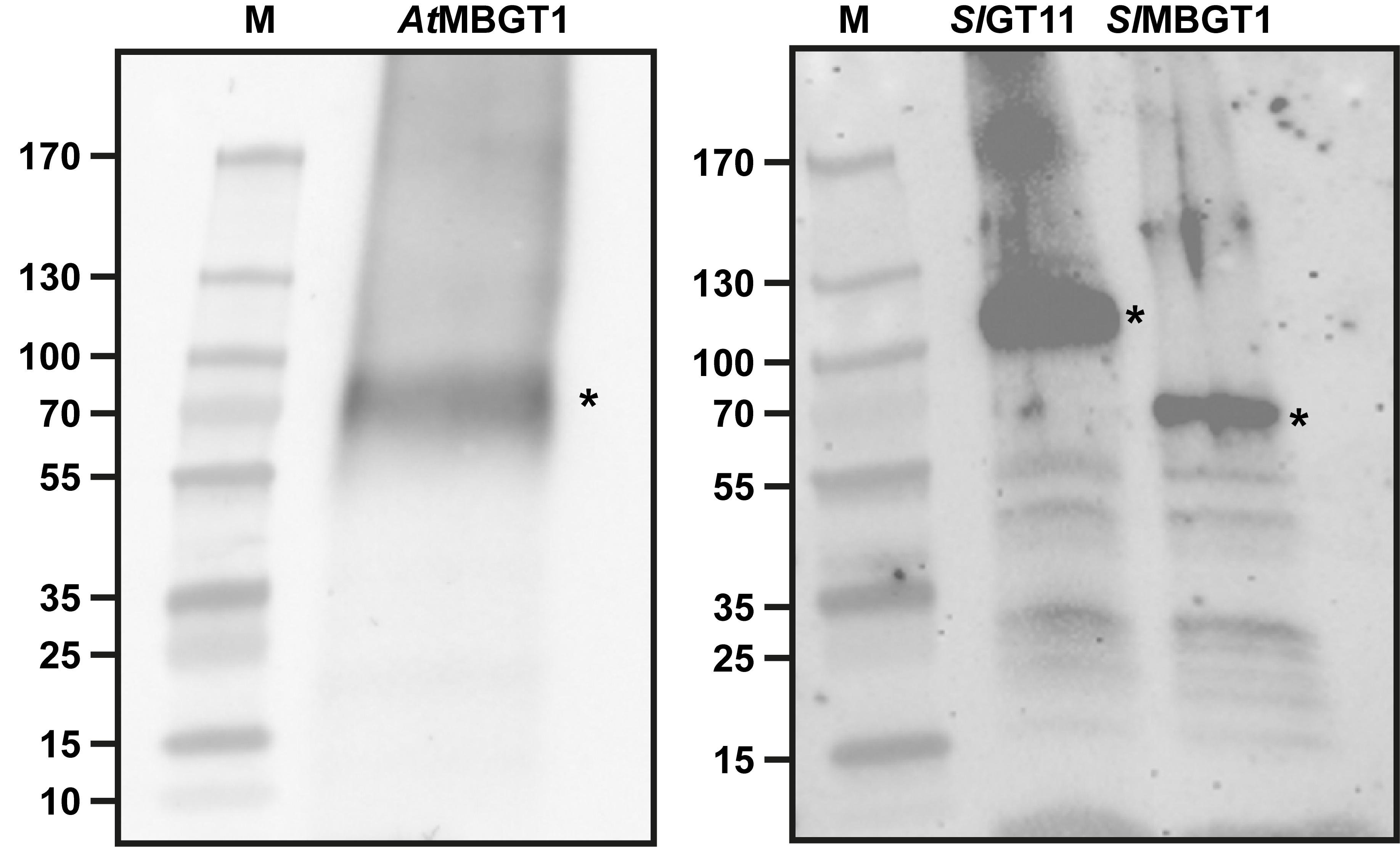


**Supplementary Data S1 Fig. 1.** Immunoblot analysis of the microsome fractions to reveal the transiently expressed proteins. *At*MBGT1 was myc-tagged, and the tomato proteins were GFP-tagged.

M: marker lane (standard proteins). *Asterisks* indicate the bands corresponding to the expressed proteins. Calculated molecular weights are: *At*MBGT-Myc, 64.9 kDa; *Sl*GT11-eGFP-6xHis, 96.3 kDa; *Sl*MBGT1-eGFP-10xHis, 89.3 kDa.


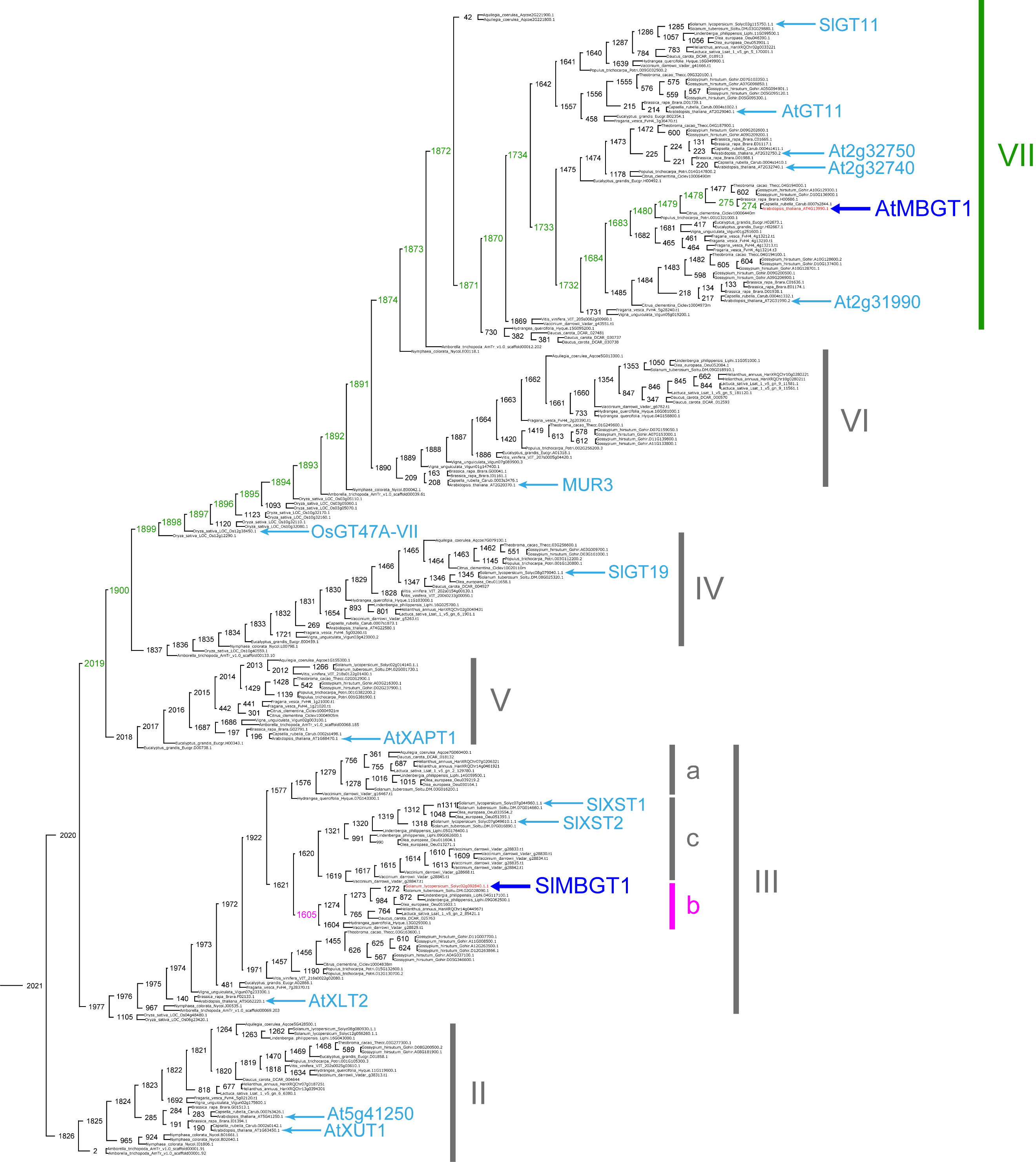


**Supplementary Data S1 Fig. 2.** Tree of the GT47A GTs. The putative asterid MBGT subclade GT47A-IIIb and the putative rosid MBGT subclade branches are labelled in pink and green, respectively. The numbers define the branchpoints between subclades, and GTs with known activity are labelled with the enzyme name.

**Supplementary Data S1 Table 1.** Nucleotide sequences used in this study.

Nucleotides in orange indicate *Bsa*I sites (GGTCTC) used for Golden-Gate cloning. Underlines indicate overhang regions used for the cloning, which become sticky ends after *Bsa*I digestion. Highlighted nucleotides in blue indicate a coding region.

| No. | Name | Purpose | Sequence |
| --- | --- | --- | --- |
| 1 | SlMBGT | Protein expression | CACTCTGTGGTCTCAAATGATGCTGTCACCTTACTCCGACGAGTCGCCGGGCGCCGATGAAGCTATGCGCAAACCTCTAAAAACTAACAACAATGATTTTCTAAAGAATGCTGTGAGTAATTTTCAGTATCAGATTTCTACTCATCCTCGTTTCTGGCTTTTTACCTTCTTCCTTTTTTTTCAACTTGTGGTGCTTATCTTTACTCGCAATTCCCCTTTCCCTTTCTCAGTTCATTCCCCACCCAGTCCTCTCCCCCAATTCCCTTCAGAAATCGATGTATTTCCCGATTTACAGACAGCCCATCACGATGCAAATGTAATTTATCCCTTCGGCGATTCTGAATGCGAGTATGGTCGGGTCTATGTCTATAATTTACCATCCAAATTTAACAAAGATTTAGCTTTGTTGACCTGTGACGATCTAGACCCATGGAAGTGGCAGTGTGGTCTTGTGACCAATGATGGATATGGAAAGAGATCGACGGAGCTCGCCGGAATCTTGCCGGGGAATCTCTCCGCAGCGTGGTATCGTACAAATCAGTTCTCATCAGAGGTAATATTTCATTACCGGCTTTTAAACTATAGATGTAGAACCAACGACCCAGAATCTGCTACGGCTTTCTACATTCCGTTCTACGCCGGACAGGCTGTTGGGAAATACCTTTGGACTGATGAAATTGAAAATCGTGATTTTCTTAGCAACAAATTACTGAAATGGGTTCAAAGGCAAAAGTATTGGAAAAAATATAAGGGTTTGGACCATTTCCTCACACTTGGTCGGATAACGTGGGATTTCCGGCGATTAGGTGACCCAGAAAAACTCTGGGGATCCTCTTTTCTAAATCGACCGCAGATGCAGAATGTTACGCGATTCACAATCGAGAAGGCTCCATGGGATGCAAACGATATCAGTGTACCTTACCCTACCGGATTCCACCCTCATTCCGAGAAAGAACTCCGAGAATGGCAAAAGTTTGCACTTTCATATAACCGCACCAGTCTATTCACCTTCATAGGGGCGGCGCGTGGGGATATCGATAGTGATTTCAGATCGAGGTTAATGAGTTACTGTAGGAACGAGTCAGACTCGTGCCGAGTTGTGGACTGTGCCGTGATTCCCTGCTCTAACGGTTCATCAGAAATCCAAAAAGCACTTCTGAGCTCAGACTTCTGTTTACAGCCCAAAGGGGACAGCTTGACTCGGCGGTCAGTTTTTGATTGCATGGTGGCTGGTTCAGTACCGGTTTTCTTCTGGAGGCGAACGGCTTACACTCAGTACCAGTGGTTTTTGCCGGAGGACCCGGGGAGTTATTCCGTGTTTATAGACCCGGAAGCAGTGAGAAATGGGACAGCTTCCATTAAGGAAATTTTGATGAGTTACAGTAAAGATCAAGTAAGGAAAATGAGGGAAAAAGTTGTAGAGACAATTCCAAGAATAGTGTATGCAAGGCCAAGTGGAGGTCTTGGGAGTGTCAAGGATGCATTTGAAATTGCAATCGAAGGAGTATTGAAGAGGGTCAAGGACGAAAACGAATGGAAGGAATACGTGGATATGGGCAGTAGTTCGTGAGACCACGAAGTG |
| 2 | SlMBGT-F | Genotyping | TTCAGAAATCGATGTATTTCCCG |
| 3 | SlMBGT-R | Genotyping | ATTAACCTCGATCTGAAATCACTATCG |
| 4 | SlGT11 | Protein expression | CACTCTGTGGTCTCAAATGGATAACTCCCTCACAACAAGATCTCGCAATAAATTTTGGTTTGTTTTCTTTTTTCTATTTGTTTCTTGGTACTTATTGCTTTATGGAATTGATTGGTCATCTTTACCTGGTTTTGTAACAACGTCACGATATGAAGTAAATTCAATTGAATCCTTTCGTCCACCTCCTCATCATGAAAATGTTCATAATGTTAGCTCCAATTTCAATGCTACAAGTGTTGATGATGATAATGCTAGCAACGAGACGACGCGATCCAAGGAGGAAGAATCACTGTCTAATGAAAATGATAATGTGGTAGTACCTGATTTGGAGGAACTCCAGAAGGAATTGGAACCTTTGTTAAGAAAAATGGAACCTCCAAAAGAAGAGAAAAAGATCGAAAAGGAAGTTGAGAAGAAGGGAGGAAAATGTGCAGGACGATATATTTATGTGGCAGAAATACCTAGTAAGTTCAATGAAATCATGTTGAAGGAATGTAAATTGTTGAATAAATGGGAAGATATGTGTCAATATTTGGTAAATATGGGGCTTGGTCCTGATCTTGGAAACCCTCAAAGGATTTTCATGAACAAAGGTTGGTATACTACGAATCAATTTTCGTTAGAAGTTCTGTTTCATAACAGAATGAAACAGTACGATTGTCTAACGAATGATTCTTCAGTAGCATCAGCAGTTTTCGTTCCATACTATTCAGGGTTCGATGTTGCTAGGTACTTGTGGGATGATTTCAACACATCAATGAGGGATGCTGGTGCAATTGAGGTCGCAAAGTTTCTCAAGGAAAAACCTGAATGGAAAACAATGTGGGGAAGAGATCATTTCATGATTGCTGGTAGAATTACTTGGGATTTTAGGAGAGGTATTGAGGAAGATTCAGCTTGGGGGAACAAGTTAATGTTGTTACCTGAGGCAAAGAACATGACTATCTTAACAATCGAATCGAGTCCTTGGAACAGGAATGATTTCGCGATTCCATATCCAACCTATTTCCATCCTTCAAGTGACAGTGATGTAGTACAGTGGCAGAACAGGATGAGGAAGTTAAGGAGGAGGGTGTTGTTTTCATTCGCGGGGGCTCCACGTCCTCAACTTGAGGACTCAATAAGAAGTGAAATCATGGAACAATGCTCAGCCACGAGGCGTAAATGCAAGCTATTGGAGTGTAAAGACTTACACAACAAATGCAACAAGCCTGAACATGTAATGAGGCTGTTTCAAAGTTCAATTTTTTGCTTACAACCCTCAGGGGATTCGTTCACTAGGAGGTCTACTTTCGATTCAATTTTGGCTGGTTGTATCCCAGTGTTTTTCACTCCTGGATCCGCGTATGTCCAATATATATGGCACTTGCCAAAAGATTACACCAAATACTCAGTGCTCATACCGGAAGATGATGTTAGGAAAAAGAAAGTGAGCATCGAAAATGTACTATCTAAAATACCAAAATCACAAGTAGCAGCAATGAGAGAAGAAGTGATAAAGCTTATACCAAATGTAGTTTATGCAGATCCAAGAACAAGATTGGAGACAGTTAAAGATGCATTTGATTTGGCAGTGAAAGGGGTTCTTGAAAGAGTGGATGTAATAAGAAAAGAGATGAGACAGGGCAAATATTCAAGTATGATATTTGATGAAGAATTTAGCTGGAAGTATCATACATTTGGGACATTACAAAAGCATGAGTGGGATAGTTTCTTTTTGAGAACCAACAAAGAGAAGTACAGTTCGTGAGACCACGAAGTG |
| 5 | SlGT11-F | Genotyping | CTCTTTCAAGAACCCCTTTCACTGC |
| 6 | SlGT11-R | Genotyping | AGTGAAATCATGGAACAATGCTCAGC |
| 7 | pHREAC-SlMBGT-F | Protein expression | CACTCTGTGGTCTCAAAAATGATGCTG |
| 8 | pHREAC-SlMBGT-R | Protein expression | CACTTCGTGGTCTCACGAA |
